# Description of pharmacists’ reported interventions to prevent prescribing errors among in hospital inpatients: a cross sectional retrospective study

**DOI:** 10.1186/s12913-021-06418-z

**Published:** 2021-05-06

**Authors:** Abdulhakim A. Alzahrani, Monira M. Alwhaibi, Yousif A. Asiri, Khalid M. Kamal, Tariq M. Alhawassi

**Affiliations:** 1 College of Pharmacy, Riyadh Elm University, Riyadh, Saudi Arabia; 2 Pharmaceutical Care Department, King Fahad Hospital, Ministry of Health, Albaha, Saudi Arabia; 3 Department of Clinical Pharmacy, College of Pharmacy, King Saud University, PO Box 2457, Office (1A229), 11451 Riyadh, Saudi Arabia; 4 Medication Safety Research Chair, College of Pharmacy, King Saud University, Riyadh, Saudi Arabia; 5 Division of Pharmaceutical, Social and Administrative Sciences, School of Pharmacy, Duquesne University, 600 Forbes Avenue, PA 15282 Pittsburgh, USA; 6 Pharmacy Services, King Saud University Medical City, Riyadh, Saudi Arabia

**Keywords:** Prescribing errors, Pharmacists related interventions, Acute care setting, Patient safety

## Abstract

**Background:**

Prescribing errors (PEs) are a common cause of morbidity and mortality, both in community practice and in hospitals. Pharmacists have an essential role in minimizing and preventing PEs, thus, there is a need to document the nature of pharmacists’ interventions to prevent PEs. The purpose of this study was to describe reported interventions conducted by pharmacists to prevent or minimize PEs in a tertiary care hospital.

**Methods:**

A retrospective analysis of the electronic medical records data was conducted to identify pharmacists’ interventions related to reported PEs. The PE-related data was extracted for a period of six-month (April to September 2017) and comprised of patient demographics, medication-related information, and the different interventions conducted by the pharmacists. The study was carried in a tertiary care hospital in Riyadh region. The study was ethically reviewed and approved by the hospital IRB committee. Descriptive analyses were appropriately conducted using the IBM SPSS Statistics.

**Results:**

A total of 2,564 pharmacists’ interventions related to PEs were recorded. These interventions were reported in 1,565 patients. Wrong dose (54.3 %) and unauthorized prescription (21.9 %) were the most commonly encountered PEs. Anti-infectives for systemic use (49.2 %) and alimentary tract and metabolism medications (18.2 %) were the most common classes involved with PEs. The most commonly reported pharmacists’ interventions were dose adjustments (44.0 %), restricted medication approvals (21.9 %), and therapeutic duplications (11 %).

**Conclusions:**

In this study, PEs occurred commonly and pharmacists’ interventions were critical in preventing possible medication related harm to patients. Care coordination and prioritizing patient safety through quality improvement initiatives at all levels of the health care system can play a key role in this quality improvement drive. Future studies should evaluate the impact of pharmacists’ interventions on patient outcomes.

## Background

The benefits of medications in relation to patient care have been one of the main focuses of the human being for centuries. Nowadays, with the obvious advances in medical science, medicines have well-known documented evidence based benefits in terms of effectively treating, managing or preventing various diseases [1]. However, medications can be a double-edged sword and medication safety has always been a major concern for both patients and healthcare professionals (HCPs) [2]. Moreover, with the growth in fast-track drug approvals in the last years coupled with the increasingly complex drug molecules and therapy regimens such as the use of biologics and other sensitive medications, drug-related complications are expected to be on the rise and require more than usual attention from all HCPs [3].

Prescribing errors (PEs), a major medication safety issue, are a common cause of morbidity and mortality, both in the community practice and in hospitals [4]. PEs are defined as “a clinically meaningful prescribing error that occurs as a result of a prescribing decision or the prescription writing process resulting in an unintentional significant reduction in the probability of treatment being timely and effective [5] or in increasing the risk of harm when compared to generally accepted practice” [6]. Despite the fact that there is variability in the documented rates of medication errors due to the utilization of various medication safety classification systems in addition to the different tools and methods of recording medication errors, PEs are nevertheless considered a common occurrence with substantially high burden [7]. Moreover, PEs are associated with higher consumption of healthcare resources and incur substantial costs to the healthcare system [8].

The occurrence of PEs in hospitals is a perennial problem and can occur at any stage of the medication process. The incidence of PEs in patient care, in part, can be attributed to the quality of care that is provided and also to the complexity of the drug regimen that is prescribed to the patient. Almost 6.5 % of morbidity and mortality in hospitalized patients have been linked to PEs, while more than half of these errors are considered as preventable [9]. Published studies have reported that PEs can also occur due to prescribing wrong medications or doses, wrong frequency, duplicate orders, and prescription of restricted medications. This problem is further compounded in critically ill patients in acute care settings who are at an increased risk for PEs given their severe health status, treatment with complex regimen, polypharmacy and constant treatment modifications [10]. Additionally, the high frequency of PEs in hospitals can also be attributed to hospital system such as inadequate medical staff training, increased work load or secondary to patient-related factors such as poor patient health literacy in addition to other various factors such as the similarly sounding medication names, and etcetera [11].

Pharmacists are considered one of the main key players in the healthcare system with other HCPs working in a multidisciplinary approach [12]. Therefore, pharmacists have an essential role in almost all medication processes and can positively impact patient care and patient safety [13]. This role can be seen in the different settings including hospital multidisciplinary teams, nursing homes as well as in the primary care settings. In terms of medication and patient safety, for instance, some studies have reported that about three-quarters of PEs can be addressed by pharmacists before reaching patients [14, 15]. Consequently, pharmacist’s role in the optimization of drug therapy and medication management, from a patient safety and quality of care point of view has always to be quantified by the level of interventions provided by pharmacists to prevent medications misadventures in addition to the other services that pharmacists provide [16].

With the advances in the pharmaceutical care filed, pharmacists have developed different patient safety approaches and have played an important role in the application of these approaches in the healthcare system to improve patient safety [17]. Their range of roles differ widely, from increasing patient knowledge through drug information or teaching patients to correctly use the medications so as to prevent adverse drug events (ADEs) during inpatient stay or post hospital discharge [17, 18]. In a clinical trial conducted in the critical care setting, the involvement of a pharmacist in patient care was associated with three- to five-fold reduction in the incidence of PEs [19]. Searching the literature shows a very few studies have addressed the nature of pharmacists’ related interventions to prevent PEs in tertiary care settings and in Saudi Arabia this issue is not well reported and thus, there is a need to explore the different interventions provided by pharmacists to minimize or prevent PEs to assess its impact on patient outcomes and pave the way for future studies that can implement the various pharmacists’ interventions and its impact on patient outcomes [20].

### Aim of study

The main aim of this study was to describe the different pharmacists delivered interventions to prevent or minimize PEs among inpatients in a hospital setting.

### Ethics approval

Ethics approval was obtained for this project through King Saud University Institution Review Board. Approval was granted on May 21, 2018: approval number [E-18-3251].

## Methods

### Study Design and setting

A retrospective cross-sectional analysis of the electronic medical records (EMRs) system of a tertiary teaching hospital was conducted to identify reported pharmacists’ interventions related to PEs. The hospital is one of the leading tertiary teaching hospitals in Riyadh, Saudi Arabia with a capacity of over 1,700 beds and more than 40 medical and surgical wards. The hospital approximate number of admissions is 50,000 new inpatients per year.

### Sample size and study period

Patients of all ages who were admitted to the inpatient care and prescribed/received a medication during the study period and had complete report and documented PEs and pharmacists’ reported interventions during the study period (April 2017 to September 2017) were included in this study. The PEs related data of all medical, surgical wards, or other wards patients was extracted. Patients from the outpatient setting or those in an inpatient setting with incomplete reported PEs and pharmacists’ interventions were excluded.

### PEs and Pharmacists Interventions Reporting process

The Electronic System for Integrated Health Information (eSiHi application) is an electronic system that allows HCPs in the study site to report all patients related data (medical, laboratory and pharmacy related data) including any medical incidents which include medication errors (MEs) data, allergies, etc…). The hospital time frame for reporting any potential MEs is 24 h for significant/sentinel event and 72 h for non-sentinel events. The data pertaining to any potential MEs to be reported includes clinical data, medication information and data related to MEs including PEs obtained by the clinical pharmacists in the different medical, surgical and other inpatients wards at the time of patient hospital admission or discharge. This part of the reporting process using the ESiHi is defined and known as the “Intervention Report”. Afterwards, once the reported PEs issue been solved by the medical team and the proper decision is taken with error got corrected, the Medication Safety Officer (MSO) who works under the hospital’s department of pharmaceutical care services (Quality and Safety Unit)traces the reports from the time received until the reported MEs get evaluated and analyzed in the system. Furthermore, to ensure accuracy of reporting, the patients’ medical record is requested and the reporter can be contacted for any clarification, when completed MEs reports get assessed and verified by the in charge MSO. A quarterly MEs analysis and report is presented to the Pharmacy and Therapeutic Committee for review and appropriate measures. This practice is performed for quality assessment to assure safe medical practice and therefore patient safety, according to the institute policy and procedures and proper actions which act hard to improve the “Just Culture” environment in addition to reporting medical related problems to the Saudi Food and Drug Authority Vigilance Department if required. The Quality and Safety Unit has an effective and consistent policy on how to handle MEs. Moreover, it gives appropriate instructions and precautions to clinical pharmacists on how to identify, report, intervene, and analyze medication errors. Also, it has a system in place for monitoring, and preventing future incidences. MSOs are highly trained with high education and training in pharmacy and medication and patient safety.

### Data Collection

All data pertaining to PEs and pharmacists’ reported interventions “Intervention Report” and data related to medication use was extracted retrospectively from ESiHi. These reports are either done by the pharmacist-in-charge of the hospital wards or in the central inpatient pharmacy, which is then retrieved by the MSO at the study site for confirmation and auditing. The extracted data was then entered by the study main investigator (AA) in an excel file designed purposefully for the study. The extracted data was cleaned, reviewed, and validated by the study co-investigator (TA). Other study team members (MA and YA) were consulted for verification or for addressing conflicting reports. The final dataset comprised of patient demographics (age, gender, and nationality), medication-related information, PEs data, and the nature of interventions performed by the pharmacists. Patients were classified into five age groups: infants (0 to 2 years), children (3 to 12 years), adolescents (13 to 17 years), adults (18 to 64 years) and older adults (65 years and higher).

### PEs Definition, categorization and pharmacists related interventions

All the PEs and comments written in the Intervention Report were reviewed and classified into 11 categories according to the hospital classification system which defines PEs as: (i) prescribing of wrong dose, (ii) prescribing of wrong medication, (iii) prescribing of duplicate therapy, (iv) prescribing of contraindicated drugs, (v) prescribing medication to patient with documented Adverse Drug Event (ADR) or Allergy, (vi) prescribing of wrong time of administration or rate, (vii) prescribing of wrong route, (viii) prescribing of wrong frequency, (ix) incomplete prescription or lack of other information, (x) unauthorized prescription (prescription of medications restricted to certain specialty by unauthorized prescriber), (xi) others.

Pharmacists’ interventions were classified into 12 different intervention types according to the hospital system: (i) preventing an adverse drug event, (ii) stopping contraindicated medications, (iii) dose adjustments, (iv) preventing drug interactions, (v) conversions from intravenous to oral formulations, (vi) discontinuation of medications with no indications, (vii) optimizing therapy monitoring (i.e. including therapeutic drug monitoring, prescriptions information quality (incomplete prescription), wrong dose and wrong frequency etc…), (viii) renal dose adjustments, (ix) approval of restricted medications, (x) therapeutic duplications prevention, (xi) recommending medications for untreated indications, (xii) others unclassified interventions.

### Medications and route of Administration classification

The medications reported in this study were classified based on the Anatomical Therapeutic Chemical (ATC) classification system. The route of administration for medications was divided into two main groups: systemic (enteral and parenteral medications) and local (topical and inhalation medications).

### Statistical analysis

Descriptive analyses were conducted using IBM SPSS Statistics (Version 21, Corp, Armonk, NY, USA). Frequency and percentage were used to describe the categorical variables. Mean and Standard deviation were used to describe continuous variables.

## Results

### Occurrence and Types of PEs

A total of 2,564 PEs and pharmacists’ interventions related to PEs among 1,565 patients were recorded in the hospital database during the study period. Patients’ age ranged from 1 to 104 years, with a mean age (± SD) of 42.72 (± 27.22) years. The highest frequency of reported PEs was among adults (18 to 64 years) had with 1,241 (48.4 %) reported PEs, while the lowest frequency of PEs reported (4.3 %) was among adolescents’ patients (13 to 17 years). PEs among other ages were also noticeable with 253 (9.9 %) PEs in infants (0 to 2 years), 257 (10.0 %) of PEs in children (3 to 12 years) and 704 (27.5 %) occurred in older patients (≥ 65 years). The reported PEs were higher in females (*n* = 1,320, 51.5 %) and among Saudi citizens (*n* = 2,117, 82.6 %) compared to males and non-Saudi, respectively. The highest rate of PEs was recorded in the medical wards followed by intensive care units (Table 1).

**Table 1:** Distribution of prescription errors according to the different hospital’s units^a^ (*n* = 2564)

	Prescription Errors	
	**No.**	**%**
**Medical wards**	1,554	60.6
Hemodialysis Unit	45	1.8
Hospital Isolation Rooms	90	3.5
Academic Staff	26	1.0
Business Center	57	2.2
Cardiac Medical Ward	38	1.5
Emergency Department	205	8.0
Internal Medicine Ward	594	23.2
Nursery Unit	8	0.3
Obstetrics and Gynecology	132	5.1
Oncology	44	1.7
Pediatric	290	11.3
Psychiatric Ward	25	1.0
**Intensive Care Unit**	614	23.9
**Surgical Unit**	390	15.2
**Radiology Department**	6	0.2

^a^Hospital units classified according to the hospital internal system as listed in this table.

The highest rate of PEs was for prescribing wrong doses (*n* = 1,393, 54.3 %) followed by PEs related to unauthorized prescriptions (*n* = 562, 21.9 %)(Table 2). Anti-infectives for systemic use and alimentary tract and metabolism were the two most common medications associated with the reported PEs (Table 3). Medications for systemic use and in particular parenteral medications had the highest rate of PEs that required pharmacists’ interventions (Table 3).

**Table 2:** Types of Prescribing Errors (PEs) (*n* = 2564)

Variables	Types of PEs	
	**No.**	**(%)**
Prescribing of wrong dose	1,393	54.3
Unauthorized prescription	562	21.9
Prescribing of duplicate therapy	279	10.9
Prescribing of Contraindicated drugs	98	3.8
Incomplete prescription/ lack of information / or require monitoring	89	3.5
Prescribing of wrong frequency	58	2.3
Prescribing of wrong route	53	2.1
Prescribing of wrong medication	18	0.7
Prescribing of wrong time of administration / Rate	7	0.3
Prescribing medication to patient with documented ADR/ Allergy	4	0.2
Failure to monitor when required	3	0.1

*ADR* Adverse Drug Reaction; *PEs* Prescribing Errors

**Table 3:** Medications’ therapeutic categories associated with PEs using ATC Classification (*n* = 2,564)

Medications’ Therapeutic Categories		N	%
Alimentary tract and metabolism (A)		466	18.2
Anti infectives for systemic use (J)		1,261	49.2
Antineoplastic and immune modulating agents (L)		16	0.6
Blood and blood forming organs (B)		267	10.4
Cardiovascular system (C )		142	5.5
Dermatologicals (D)		38	1.5
Genitourinary system and Sex hormones (G)		8	0.3
Musculoskeletal system (M)		51	2.0
Nervous system (N)		216	8.4
Pituitary, hypothalamic hormones, systemic hormonal preparations, and insulin analogues (H)		23	0.9
Respiratory System (R )		71	2.8
Sensory Organs (S)		4	0.2
Various (V)		1	0.1
**PEs according to Route of Administration**			
**Systematic**			
Enteral	1,039		40.5
Parenteral	1,498		58.0
**Local**			
Topical	4		0.2
Inhalation	23		0.9

*ATC* The Anatomical Therapeutic Chemical (ATC) Classification System; *PEs* Prescribing Errors

### PEs and Pharmacists’ Interventions

Pharmacists’ interventions to prevent PEs were categorized into 12 types. The highest observed pharmacists related interventions to prevent PEs were related to dose adjustments (*n* = 1,128, 44 %), followed by approval of restricted medications (*n* = 562, 21.9 %), prevention of therapeutic duplication (*n* = 281, 11.0 %), renal patients dosing (*n* = 249, 9.2 %), and optimizing therapy monitoring (*n* = 179, %6.8 (Table 4).

**Table 4:** Types of Pharmacists Interventions Related to PEs (*n* = 2564)

Type of Intervention	No.	(%)
Dose adjustment	1,128	44.0
Restricted medication approval	562	21.9
Therapeutic duplication	281	11.0
Renal dosing interventions	249	9.7
Optimize therapy monitoring	174	6.8
IV to PO conversion^a^	53	2.1
Contraindicated drugs	48	1.9
Drug interaction	46	1.8
Untreated Indications	9	0.4
No indication for therapy	8	0.3
Adverse Drug Event	4	0.2
Others	2	0.1

^a^I*V*  Intravenous; *PEs* Prescribing Errors; *PO* Oral

## Discussion

The present study retrospectively identified PEs and the different pharmacist’s related interventions in an inpatient setting in a large tertiary teaching hospital in Saudi Arabia. A total of 2,564 PEs were recorded during a six-month period affecting 1,565 patients in a ratio of 1.7 pharmacist interventions for encountered PEs per a patient.

There is a high burden of PEs on the healthcare system in Saudi Arabia. A systematic review of PEs in Saudi Arabia (both community and hospital settings) reported the PEs rates vary from 7.1 to 94 % with a median error rate of 32 % [21]. Another systematic review evaluating PEs in patients admitted to a hospital found median rates of 52 PEs per 100 admissions and 24 PEs per 1,000 patient days [22]. There seem to be a huge discrepancy in the prevalence of error rates including their method of estimation. This is mainly due to variability in study settings, techniques used to detect PEs [23], and also the variability in PEs definitions applied by the different studies [24]. This could potentially explain the difference in the prevalence of PEs recorded in Europe compared to those in the Middle East [25]. Therefore, there has been a growing interest in the PEs area in terms of standardizing the definitions of PEs encountered in Saudi Arabia hospitals. As a result, a recently published study tried to operationalize the different PEs in the Saudi Arabia hospital practice [24]. The availability of a standardized list of PEs to the pharmacists similar to the one applied by the study site may improve their participation in shared decision-making before medications are administered to patients in the acute care units [26]. It can also serve as a valuable resource for any future research related to PEs [24].

The present study showed that the PEs was more common in the medical wards as compared to the surgical ward. This is has been noticed in other studies which have explained the higher rate PEs in medical wards due to the higher proportion of drug prescribing being on admission [27]. In this study, pharmacists recorded 12 different type of PEs, where most of the PEs recorded were related to prescribing wrong doses. Also it is worth mentioning that among the reported PEs unauthorized prescription was fairly high and ranked second after prescribing of wrong dose representing half of the whole errors in the study population [7]. This is consistent with the findings of another study conducted in a university hospital in Riyadh, Saudi Arabia in 2011, which reported that the leading PEs were associated with wrong dose and wrong strength [28]. Our finding is consistent with a study that was conducted in the inpatients setting in a teaching hospital in UK which reported that most of the prescribing errors (54 %) were associated with choice of dose [23]. A systematic review of nine studies that involved 3,507 records reported that the top type of prescribing errors was wrong dose which ranged between 31and 91 % [22]. It is worth to mention that among the different age groups, adult patients experienced the highest PEs rate. This finding is not consistent with other published studies which found that the most affected group with PEs were elderly patients of age 65 and older [29, 30]. The difference could be attributed to the larger range of adults group included in our study, which included both middle age and young adults which reflect the dominance of young adults in Saudi Arabia where approximately two-third the population age between 15 and 64 years.

The pharmacists’ interventions for adjusting doses were the most prevalent intervention followed by restricted medication approval where the most commonly involved class of medications associated with these interventions were anti-infectives for systemic use and alimentary tract and metabolism. Surprisingly, this study reported that only 0.2 % of the pharmacists interventions were related to ADEs and this finding was in contrast with those reported by the Harvard Medical Practice Study which reported that 19 % of the interventions are related to adverse events in hospitals and emergency department settings [31]. This difference can be attributed to the fact that the Harvard study was conducted in an emergency department, where most of ADEs were detected [19], while the present study included all patients admitted to the hospitals. Despite the different interventions required by pharmacists to prevent PEs, current evidence demonstrate that hospital pharmacist’s participation in inpatient care can result in significant decrease of avoidable PEs [23]. Yet, limited information is available on pharmacists’ interventions within the inpatient setting, especially in Saudi Arabia [26, 28]. A meta-analysis of four controlled trials investigating the impact of pharmacist’s interventions on PEs revealed that pharmacists’ interventions have a significant impact in reducing PEs [32]. This study results were consistent with the findings reported in the meta-analysis in terms of reduction avoidable PEs.

This study investigated the classes of medications as it related to the incidence of PEs. It was found that systemic use of anti-infective agents was a leading reason for PEs with almost half the errors belonged to this class. This finding is consistent with a recent data supporting that PEs were mostly associated with the orders for antibiotics [33], while other drugs such as antiepileptics, had less frequent errors. Another study conducted in 20 hospitals in UK that included 26,019 prescriptions show that the most of PEs were in parenteral administration of drugs, however, they reported that cardiovascular and endocrine medications were the most common classes associated with PEs [34].

This study utilized a large sample size, and tried to investigate an important research question that can lead to future research to improve the current pharmacy practice towards safer medication use and preventing or minimizing PEs among hospital setting patients. Nevertheless, there are some inherent limitations in this study. First, although the study was conducted in a single tertiary care hospital that is considered one of the largest hospitals nationally, yet this may limit the generalizability of the study results to other practices within Saudi Arabia making the need for future multicenter studies that included different hospitals with different practices is warranted. Second, the PEs were extracted from the medical records and it is possible that some PEs may not have been recorded therefore future prospective approaches worthy to be conducted. This also necessitate the conduction of future studies to evaluate the prevalence of PEs and therefore the impact of pharmacists interventions on reducing PEs and medications related adverse events from reaching patients. Finally, both the PEs and pharmacists’ interventions were operationalized for the study, and yet, there may be variations among the hospital pharmacists in how they provided their interventions and the quality of reporting. However, this study still provides critical information related to patient safety and the significant role of hospital pharmacists in preventing avoidable PEs from reaching patients.

## Conclusions

In this study, PEs occurred commonly and pharmacists’ interventions were critical in preventing possible harm to patients. There is a need for coordination of care among different HCPs to prioritize patient safety through quality improvement initiatives at all levels of the health care system where pharmacists can play a key role in this quality improvement drive. Future studies should evaluate the impact of pharmacists’ interventions on patient outcomes.

## Data Availability

The datasets used and/or analyzed during the current study are available from the corresponding author on reasonable request.

## Abbreviations

ADEs: Adverse Drug Event
ADR: Adverse Drug Reaction
ATC: Anatomical Therapeutic Chemical
ESiHi: Electronic System for integrated Health information
HCPs: Healthcare Professionals
IRB: Institutional Review Board
MSO: Medication Safety Office
PEs: Prescribing Errors
SPSS: Statistical Package for the Social Science
